# Progress in Ophthalmology

**Published:** 1901-04-27

**Authors:** 


### Progress in Ophthalmology.

**Transplantation of Cornea and Sclera.**—Wolffberg[1] quotes a case of this which is almost a curiosity. The patient, it appears, lost the right eye from an injury in childhood, and lately injured the other by a blow causing dislocation of the lens, cataract, and irido-cyclitis. The cornea became atrophied, and after extraction of the lens only perception of light remained. The eye of a sparrow was used in this operation. The front part of the eye, that portion in front of the iris, was cut away, which practically means the cornea with a narrow ring of sclera surrounding it. As it did not seem a good plan to bring the anæmic sclera of the sparrow in contact with the anæmic posterior part of the blind eye, an artificial hyperæmia was induced, by undermining the conjunctiva all round the cornea, passing a suture after the manner of a purse string along its edge, and by pulling on this covering the atrophied cornea by the chemotic vascularised conjunctiva. On the following day the cornea was trephined through its entire thickness without loss of vitreous. The removed portion measured 3½ millimetres in diameter. The sparrow's cornea, with its surrounding ring of sclera, was introduced into this opening and its edges tucked away under the overlapping conjunctiva. Scarcely any reaction ensued. The sutures were removed at the end of the fifth day. The transplanted sclera soon lost its opaqueness and became as transparent as the cornea. He states that at the end of a month the patient had enough vision left to see her way about a well lighted room. This is all very well for a month or so: we ourselves have often seen the operation performed in the Vienna Clinic, the only difference being that the transplanted cornea was taken either from a rabbit or a human eye, which had to be enucleated for some other reason. In no case was the whole cornea used there, merely a small piece about 2½ mm., and this may, of course, account for its becoming opaque so soon. Still, one would like to hear how this sparrow's cornea had behaved at the end of six months.

**An Operation for the Relief of Stoppage of the Tear Passage, Abscess of the Lachrymal Sac, &c.** Dr. Erasmus Pond[2] mentions a new method for the relief of epiphora, &c. The present methods are all so unsatisfactory that one hails with delight any new procedure which may procure relief. Briefly he writes as follows: The object of this operation is to establish a permanent opening into the nose and to give in this way free drainage to the lachrymal sac, thus relieving epiphora and obviating any danger of an abscess in one operation. The operation is very simple. A long silver probe, with one end blunt and the other end having an eyelet large enough to carry a coarse silk string, is threaded and passed through the canal into the nose where the end is seized with a pair of forceps and drawn out through the nostril. The probe is then unthreaded and the string left in this position with the ends tied together. The string is left one week, being drawn through the nose two or three times a day. He usually, when tying, makes quite a large knot and when this is pulled through the canal, it makes a good large opening. He has used this operation for the past two years with gratifying success. The canaliculus may be slit or not according to the judgment of the operator. He has done most of these operations under cocaine, but has sometimes used ether. There is no pain after the first operation, and the string may be turned without causing any hurt. In abscess of the sac, the string will give good drainage and so save the necessity of an opening in the cheek.

**Eclipse Blindness.**—At the January meeting of the Ophthalmological Society a very interesting account was given on a case of eclipse blindness by Dr. Batten.[3] The subject always crops up after an eclipse has taken place and no one seems to know exactly why. In this particular case there was thrombosis of a retinal artery and hæmorrhage into the vitreous. It appeared the patient had watched the eclipse of May 28th, 1900, without any other protection than that afforded by screwing up her eyes and looking between her fingers held closely together. Immediately afterwards she noticed that things looked black and the next morning she could only see "portions of things." She was first seen on June 6th and was found to have lost the lower half of the field of the left eye. The vision was $\frac{6}{18}$. Above the optic disc a white patch was seen (probably an absorbing hæmorrhage), while the hazy and œdematous retina obscured the view of the disc and retinal vessels. The œdema increased till June 20th and then rapidly cleared, ultimately leaving a patch of choroido-retinitis above the disc. One of the upper retinal arteries was occluded, and the others were reduced in size. The vitreous opacities cleared and the macula was unaffected. The vision eventually improved to $\frac{6}{12}$ in the injured eye, in the other it was normal. The patient was in good health and no other cause could be found for the injury except exposure to direct sunlight. Several other cases were quoted in which there were nearly always central scotomata remaining as a result. Dr. G. A. Berry thought the public was not sufficiently aware that blue glasses were worse than useless when looking at the sun and bright lights—it was the blue and violet rays that did all the damage. In Russia red or yellow glasses were employed.

**The Older and Newer Mydriatica in Ophthalmology.**—Dr. Schulze,[4] who appears to have given a good deal of attention to this matter, says: A modern mydriatic of short effective duration is euphthalmine hydrochloride. In a 5 per cent. solution it was an effective mydriatic. Used as an instillation, it caused very mild burning, not more that a cocaine solution would. The effect began after 10 to 15 minutes, gradually increased, and had in 60 to 80 minutes attained the highest intensity. Maximal mydriasis then existed, of about eight mm. and more. The reaction to light was extinguished. It caused at the same time an accommodation paralysis, which was, however, less than that caused by homatropin. The maximum of the effect lasted only a short time. After two to three hours the inconvenience from the paralysis of accommodation disappeared, and in from four to five hours the effect had almost passed away. Comparative observations, with 1 per cent. homatropin and 5 per cent. euphthalmin solutions in the two eyes of one and the same individual, proved that homatropin mydriasis began as a rule somewhat quicker, perhaps three to five minutes earlier, than that of euphthalmin. The euphthalmin mydriasis was ended after three to five hours, while that of homatropin was still observable the next day. The intra-ocular pressure, according to Schneider, who tried the remedy in glaucoma, is not increased. No disagreeable by-symptoms were observed. Euphthalmin, therefore, acts only inappreciably slower than homatropin, while in other respects it is equally effective. Euphthalmin has, however, the advantage over homatropin, inasmuch as its action is of shorter duration, and it causes less disturbance of the accommodation. Over cocaine it has the advantage that it by no means alters the corneal epithelium. We consider it, therefore, a very excellent mydriatic for diagnostic pupil dilatation, while we would prefer homatropin for objective refraction determination on account of its greater effect in the paralysis of accommodation.

**Mental Disturbances after Operations on the Eye.**—Dr. Posey[5] thinks that these disturbances are more common after cataract than after other operations. They take the form of a delirium, appearing on the evening of the third day and continuing up to a week. The ultimate success of the operation is not affected as a rule. It usually occurs in persons of advanced age. The intensity of the delirium varies, and it is most marked at night. The psychoses met with have been divided into four groups. (1) Hallucinatory mental derangement in non-alcoholics; (2) simple confusion of ideas in senile subjects—hallucinations are not present and dementia not rarely follows; (3) delirium in alcoholics; (4) confusion of ideas due to inanition, occurring in extremely marasmatic individuals and followed by death. Sixteen cases occurred in the Wills Eye Hospital during four years, in which time 770 extractions of cataract took place. The delirium has been attributed to many causes without much reason, and Dr. Posey is of opinion that the real cause is largely psychic and due to the preoccupation on the part of the patient prior to, and after the operation, aided by the constrained position and unusual stillness of the surroundings. The condition should be treated, he thinks, by the free and repeated administration of chloral and bromides. Constant oversight and tactful nursing are essential, and amelioration often follows the presence of a proper person at the bedside. He deprecates the removal of the bandage from the unoperated eye, as, he says, this does no good, but there we are not prepared to agree with him, as we have seen several cases in which this was followed by a good result.

**The Electric Treatment of Chronic Glaucoma.**—Dr. E. M. Valude[6], of Paris, has recently published an account of a new method of treatment in chronic glaucoma. The results of re-section of the cervical sympathetic led Dr. Valude to experiment whether it were not possible to reduce the tension of a glaucomatous eye by the sedative action of electricity on the sympathetic, and by destroying temporarily the excitability thereof. The method employed is to apply the negative electrode (about 300 sq. c.m. in size) to the nape of the neck. The positive electrode (about 8 or 10 c.m. long by 2 or 3 c.m. broad) is carefully protected by the ordinary coverings and an additional layer of cottonwool soaked in hot water, and applied to the anterior edge of the sterno-mastoid in its whole length. It is secured by a bandage. The current used is one of 20 milliamperes, the electro-motive force of 20 volts, and is allowed to pass for 15 minutes; the treatment to take place once every three days. He quotes six cases, in all of which very material benefit resulted from the treatment, and concludes that in ordinary simple chronic glaucoma, if myotics have done all they can, and the disease still progresses, this form of galvanisation with the sedative effect of the positive pole, should be tried before any operative interference is suggested, especially before the sympathetic cervical ganglion is excised—an operation which is difficult to perform, and has even in the most skilled hands been followed by the death of the patient from cardiac disturbance.

[1] Quarterly Med. Jour., Feb. 1901. [2] Med. Rec., Feb. 2, 1901. [3] Lancet, Feb. 9, 1901. [4] Therapist, Feb. 15, 1901. [5] Med. Chron., Jan. 1901. [6] Treatment, Nov. 1900.

